# EUS-guided blood patch delivery during liver biopsy: nature’s gel foam

**DOI:** 10.1016/j.vgie.2021.06.006

**Published:** 2021-07-22

**Authors:** Piotr Sowa, Jennifer Kolb, Jason Samarasena, Kenneth J. Chang

**Affiliations:** Digestive Health Institute, Chao Family Comprehensive Digestive Disease Center, University of California, Irvine

## Abstract

Video 1 Case 1 EUS liver biopsy with FNA needle complicated by persistent blood flow within biopsy tract, which is successfully treated with blood patch technique. Case 2 EUS liver biopsy with FNA needle complicated by persistent blood flow within biopsy tract, which is successfully treated with blood patch technique.

Case 1 EUS liver biopsy with FNA needle complicated by persistent blood flow within biopsy tract, which is successfully treated with blood patch technique. Case 2 EUS liver biopsy with FNA needle complicated by persistent blood flow within biopsy tract, which is successfully treated with blood patch technique.

## Introduction

EUS-guided liver biopsy is emerging as an alternative form of tissue acquisition to percutaneous or interventional radiology–guided liver biopsy. Recent studies have shown adequate specimen acquisition and an enhanced safety profile. EUS-guided liver biopsy has been found to be extremely safe. The most common adverse events are mainly due to bleeding events. Unfortunately, there are limited endoscopic interventions available to prevent bleeding during liver biopsy.

Autologous blood patches have been used for hemostasis since the 1960s for management of cerebrospinal fluid leaks during lumbar puncture.^1^ Recent studies have also shown efficacy with blood patch pleurodesis for alveolaopleural fistula closure.^2^ With this in mind, we have developed a novel approach to facilitating hemostasis to prevent active bleeding during EUS-guided liver biopsy by delivering a blood patch through the FNB needle into the biopsy tract before needle extraction. This results in cessation of bleeding within the needle tract, thereby preventing post–liver biopsy hemorrhage.

## Procedure technique

**Figure 1:**
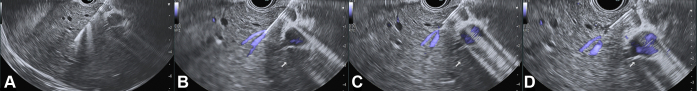
Blood patch delivery. **A,** The 19-gauge FNB of right liver lobe. **B,** Active flow within liver biopsy tract. **C,** Blood patch delivery into needle tract. **D,** No further flow within needle tract.

EUS-guided liver biopsy is performed with a 19-gauge FNB needle. With this particular needle, we perform 1 pass and 3 actuations (see Fig. 1). Before removing the needle from the liver, eFlow color Doppler is used to identify potential bleeding within the liver biopsy tract. If eFlow persists after waiting 2 to 3 minutes, the blood patch technique is performed. While the needle is within the biopsy tract, the FNA stylet is inserted into the needle, pushing out about 25% of the distal needle contents. This “blood patch” acts as a mechanical barrier to facilitate hemostasis.

## Case summary

In both cases, we encountered active flow within our liver biopsy needle tract after liver biopsy. After waiting 2 to 3 minutes, we saw persistent flow within the biopsy tract, raising concerns for post–liver biopsy hemorrhage. We then performed the blood patch technique by pushing the distal 25% of the needle contents into the needle tract. We show that this maneuver halted flow toward our needle, thereby avoiding post−liver biopsy hemorrhage and allowing us to safely remove the needle from the liver. Both patients did well after this procedure, and no adverse events were encountered.

## Conclusions

Post−liver biopsy bleeding is a known adverse event after liver biopsy. Currently, there are no endoscopic interventions available to prevent post−liver biopsy bleeding. We describe a technique in which a blood patch is delivered into the biopsy tract to facilitate hemostasis and prevent post−liver biopsy hemorrhage (Video 1, available online at www.giejournal.org).

## Disclosure

*Dr Chang is a consultant for Olympus, Apollo Endosurgery, Cook Medical, Erbe, and Medtronics and receives educational grants from Olympus, Apollo Endosurgery, and Cook Medical. Dr Samarasena is a consultant for ConMed, Cook Mauna Kea, Medtronics, Olympus, US Endoscopy, and Pentax; has ownership in DocBot; and receives educational grants from ConMed and Cook. All other authors disclosed no financial relationships.*

## Supplementary data

Video 1 Case 1 EUS liver biopsy with FNA needle complicated by persistent blood flow within biopsy tract, which is successfully treated with blood patch technique.
